# Inhibition of Sat1 alleviates myocardial ischemia-reperfusion injury through regulation of ferroptosis via MAPK/ERK pathway

**DOI:** 10.3389/fphar.2024.1476718

**Published:** 2024-11-13

**Authors:** Zhou Liu, Hongjin Chen, Yingnan Song, Kaiyuan Chen, Sisi Pan, Siyuan Yang, Deqin Lu

**Affiliations:** ^1^ School of Basic Medical Sciences, Guizhou Medical University, Guiyang, Guizhou, China; ^2^ Translational Medicine Research Center, Guizhou Medical University, Guiyang, Guizhou, China; ^3^ Division of Cardiovascular Surgery, The Affiliated Hospital of Guizhou Medical University, Guiyang, Guizhou, China; ^4^ Division of Cardiovascular Surgery, The Affiliated Hospital of Zunyi Medical University, Zunyi, Guizhou, China

**Keywords:** SAT1, myocardial ischemia-reperfusion injury, ferroptosis, bioinformatic analysis, MAPK/ERK

## Abstract

**Introduction:**

Myocardial ischemia-reperfusion injury (MIRI) is a prevalent complication in patients with myocardial infarction. The pathological mechanism of MIRI remains elusive. Ferroptosis plays a critical role in MIRI. This study aimed to investigate the role of spermidine/spermine N1-acetyltransferase 1 (Sat1) in MIRI by regulation of ferroptosis.

**Methods:**

Rats and H9C2 cells were used to perform MIRI model. The extent of myocardial damage and associated pathological changes were evaluated. Protein expression was detected by western blot. Then we observed the mitochondrial morphology and measured cell viability and damage. The levels of lipid peroxide and glutathione were measured, and lipid reactive oxygen species (ROS) was quantified. Differentially expressed genes (DEGs) in MIRI were analyzed. Moreover, to explore the role of Sat1 in MIRI, this study utilized adeno-associated virus 9 and lentiviral transduction to modulate Sat1 expression in rats and H9C2 cells, respectively. The transcription factor that regulates Sat1 expression was predicated. Luciferase reporter gene experiment was conducted to reveal the potential sites of Sox2 binding to Sat1.

**Results:**

This study revealed that ferroptosis was involved in MIRI. Through bioinformatic analysis, Sat1 was identified as a significant gene in MIRI, which has been reported as an inducer of ferroptosis. Our results showed that Sat1 expression was significantly increased in MIRI. Next, the study showed that inhibition of Sat1 alleviated MIRI by suppressing ferroptosis in vivo and *in vitro*, and over-expression of Sat1 promoted MIRI via activation of ferroptosis. Furthermore, Sat1 and its interacting genes were enriched in several signaling pathways, including ferroptosis and the MAPK signaling pathway. The results showed that Sat1 regulated MIRI through ferroptosis via MAPK/ERK pathway. Moreover, it is found that Sox2 can suppress Sat1 expression at the transcriptional level. The potential binding site was TAACAAAGGAA.

**Conclusion:**

In sum, this study demonstrated Sat1 expression was increased in MIRI, inhibition of Sat1 can alleviate MIRI by regulating ferroptosis via MAPK/ERK pathway, and Sat1 was negatively regulated by Sox2. These findings suggested that Sat1 may serve as a potential therapeutic target for the treatment of MIRI.

## Introduction

Myocardial ischemic disease caused by coronary artery disease is one of the major causes of morbidity and mortality worldwide (Arrigo et al., 2021). Although timely reperfusion is the most effective therapy for rescuing myocardial infarction, it can also result in additional cardiomyocyte death and exacerbate cardiac dysfunction, leading to a condition known as myocardial ischemia-reperfusion injury (MIRI) (Zhou et al., 2021). Several mechanisms have been testified in the development of MIRI, including inflammation, programmed death, mitochondrial dysfunction, calcium overload, insulin resistance (Chen KY. et al., 2023; Zhang et al., 2019), and endothelial cell dysfunction (He et al., 2022; Toldo et al., 2018; Zhao et al., 2021). Despite efforts to develop interventions based on these mechanisms for MIRI, clinical therapeutic effects have been unsatisfactory, highlighting the need for further research to understand the underlying mechanism of MIRI better.

Ferroptosis is a type of iron-dependent cell death caused by a disturbance in the antioxidant system and lipid peroxidation, as well as elevated levels of reactive oxygen species, ultimately resulting in cell death (Dixon et al., 2012; Jiang et al., 2021). Recently, accumulating evidence has demonstrated that ferroptosis plays a significant role in MIRI. Studies showed that, after ischemia/reperfusion, myocardial infarction patients and mice displayed higher concentrations of iron (Baba et al., 2018). Resveratrol could alleviate MIRI by reducing iron accumulation through decreased transferrin receptor 1 (TFR1) expression, which plays an important role in ferroptosis (Li et al., 2022; Tang et al., 2021a). Inhibition of ferroptosis in MIRI can reduce the accumulation of ROS, glutathione, lipid peroxidation, and iron, leading to decreased cardiomyocyte death and improved cardiac function (Ma et al., 2020). Additionally, Ferrostatin-1, an inhibitor of ferroptosis, can reduce myocardial infarction size by increasing glutathione peroxidase 4 (GPX4) expression and reducing the accumulation of iron and ROS via inhibition of endoplasmic reticulum stress (Li et al., 2020). Interestingly, Tang et al. reported that only after MIRI rather than myocardial ischemia, the expression of GPX4 was significantly reduced, and malondialdehyde and iron were significantly increased (Tang et al., 2021b). These results collectively indicate that ferroptosis plays a key role in the pathogenesis of MIRI, emphasizing the importance of identifying new targets for regulating ferroptosis.

The enzyme Spermidine/Spermine N1-Acetyltransferase 1, which is encoded by the Sat1 gene, is primarily localized in the cytoplasm. The protein encoded by the Sat1 gene belongs to the acetyltransferase family, and is considered as the rate-limiting enzyme in the metabolism and breakdown of polyamines (Pegg, 2008). Through catalyzing the acetylation and conversion of spermidine and spermine to putreamine via the acetyl-CoA pathway, Sat1 participates in the regulation of cellular polyamine content (Casero et al., 2018). Abnormal polyamine metabolism and dysregulation of Sat1 expression have been linked to a range of diseases and pathological conditions, including cancer (Holbert et al., 2022), metabolic syndrome (Castoldi et al., 2020), MIRI (Tong et al., 2018) and ferroptosis (Ou et al., 2016). Polyamine levels have been reported to decrease significantly after MIRI, and insulin has been shown to improve cardiac function and reduce myocardial infarction injury by increasing polyamine levels (Tong et al., 2018). In rats, levels of spermidine and spermine were observed to gradually decrease over time after MIRI (Han et al., 2009). Supplementation with spermine has been found to reduce LDH levels and ROS accumulation, thereby alleviating myocardial damage (Zhao et al., 2007). Moreover, the activation of Sat1 has been shown to promote lipid peroxidation, rendering cells more susceptible to oxidative stress and contributing to the development of ferroptosis (Ou et al., 2016). Astrocytic ferroptosis has been shown in Alzheimer’s disease, and single-cell sequencing studies revealed that Sat1 may be a key molecule involved in the regulation of ferroptosis (Dang et al., 2022). Therefore, it is plausible to hypothesize that Sat1 may also play an important regulatory role in MIRI through the mechanism of ferroptosis.

In this study, the induction of ferroptosis in MIRI was observed. A significant increase in Sat1 expression level was demonstrated. The enrichment analysis revealed that the ferroptosis and the MAPK signaling pathways were enriched in Sat1-interacting genes. Furthermore, inhibition of Sat1 has alleviated MIRI via regulating ferroptosis *in vitro* and *in vivo*, and overexpression of Sat1 promoted MIRI via activation of ferroptosis. Moreover, MAPK/ERK pathway was demonstrated to be involved in ferroptosis and MIRI, and Sat1 can regulate the activation of MAPK/ERK pathway. Besides, Sat1 expression was regulated by Sox2 at transcriptional level. These results collectively providing novel insights into the management of MIRI.

## Methods

### Establishment of MIRI rat model

**FIGURE 1:**
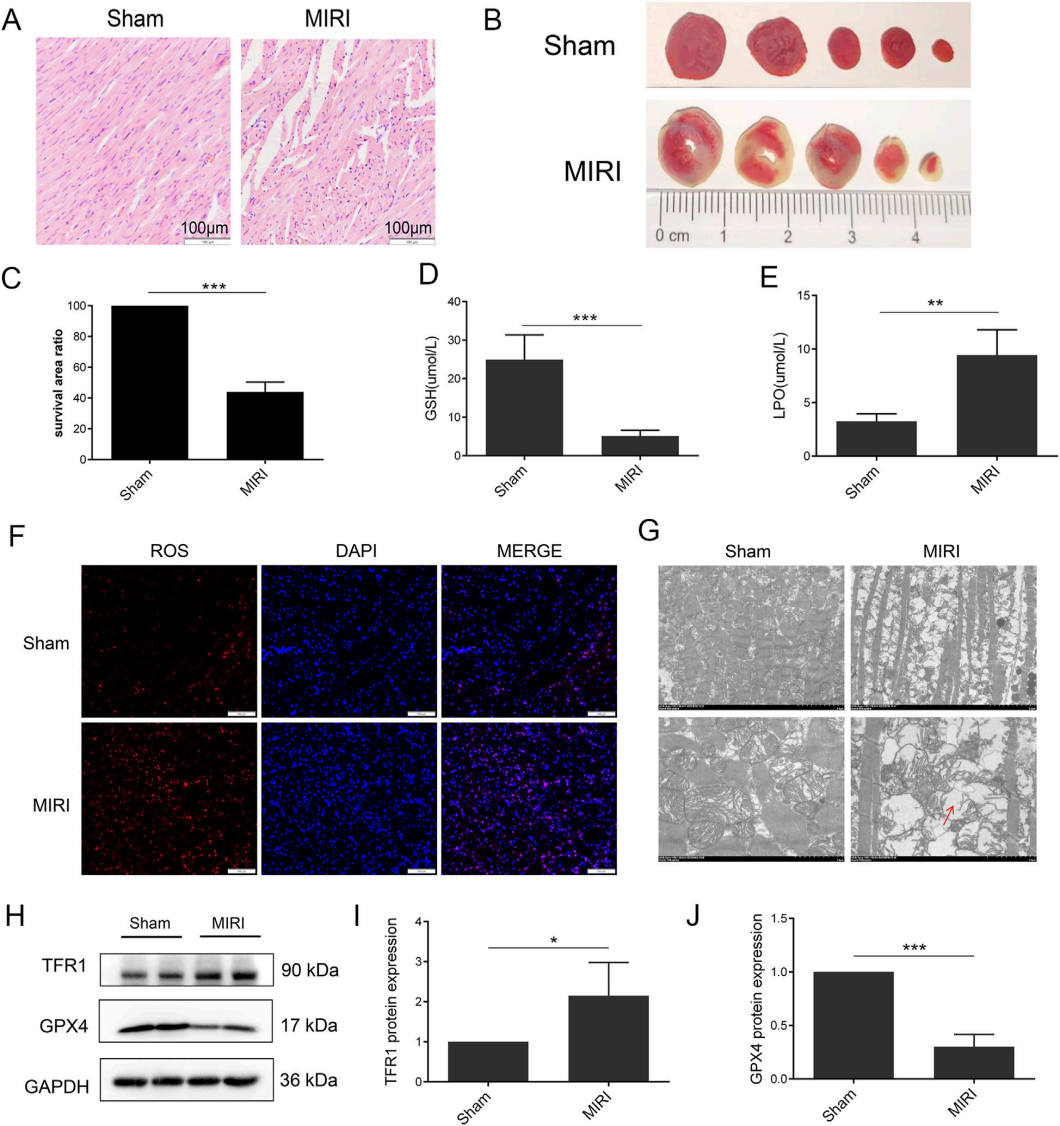
Ferroptosis was induced in MIRI. **(A)** HE staining of heart tissue; **(B)** TTC staining of heart tissue; **(C)** Quantification of infarct size. **(D)** Serum level of GSH; **(E)** Serum level of LPO; **(F)** ROS staining of heart tissue; **(G)** Mitochondria morphology of heart tissue; **(H)** Protein expression of TFR1, GPX4 and GAPDH in rat; **(I, J)**. Quantification of relative protein expression level of TFR1 and GPX4 (n = 4 for each group). Significance levels are denoted by *P < 0.05; **P < 0.01; ***P < 0.001.

Sprague–Dawley (SD) rats weighing 250–300 g were purchased from Guizhou Medical University (license number: SCXK-2019-0014). All rats were housed in a standard environment with a temperature of 21°C–25°C, a humidity range of 40%–60%, and a 12 h light/dark cycle. All rats were provided with free access to food and water throughout the experiment. After anaesthetizing the SD rats with an intraperitoneal injection of pentobarbital (50 mg/kg), a thoracotomy was performed at the left 3-4 intercostal space to expose the heart. The left anterior descending (LAD) was ligated with 6–0 silk suture to induce myocardial ischemia. The successful induction of myocardial ischemia was confirmed by observing an elevated ST segment in the electrocardiograph and a color change in the apical region to white. After 45 min of myocardial ischemia, the ligature was loosened for reperfusion. After 1 h of reperfusion, the rats were then euthanized, and the hearts and blood were collected for further analysis. For cardiac function measurements, M-mode echocardiography was used, and left ventricular internal diameter at end-diastole and left ventricular internal diameter at end-systole was measured to calculate the value of ejection fraction (EF). To suppress Sat1 expression *in vivo*, 44 μL of Adeno-associated Virus 9 (AAV9, 3.42E+13 vg/mL) and 56 μL saline was mixed and injected into rats through tail vein at a dose of 1.5 × 10^12^ vg. The target sequence of Sat1 was ACC​TAT​GAC​CCA​TGG​ATT​GGC. For the experiments in Figure 1, a total of 16 male rats were randomly divided into 2 groups: Sham group (n = 8) and MIRI group (n = 8). For the experiments in Figure 3, a total of 32 male rats were randomly divided into 4 groups: Sham group (n = 8), MIRI group (n = 8), AAV9-sh-Scr + MIRI group (n = 8), AAV9-sh-Sat1+MIRI group (n = 8). For each group, 4 rats were only used for TTC, the left 4 rats were used for experiments of HE, WB, ROS staining, and Transmission Electron Microscopy.

### Cell culture and establishment of OGD/R model

An embryonic cardiomyocyte cell line, H9C2, was purchased from the Cell Bank of the Chinese Academy of Sciences, Shanghai, China. The H9C2 cells were cultured in Dulbecco’s modified Eagle’s medium (DMEM) supplemented with 10% fetal bovine serum and 1% penicillin/streptomycin. Cells were incubated under normal conditions at 37°C with 95% air and 5% CO_2_. To induce the OGD/R model, H9C2 cells were initially grown to 80%–90% confluency, switched to glucose-free and serum-free DMEM, and placed in an incubator with 5% CO_2_, 94% N_2_, and 1% O_2_ at 37°C for 3 h. After 3 h, the cells were returned to a normal medium and incubated under normal conditions for 30 min. 40 μL HiTransG, 2 μL lentivirus (1E+9TU/mL) and 958 μL culture medium were mixed and added to each plate in six-well plate to perform lentivirus infection.

### Hematoxylin and eosin staining

The left ventricular heart tissue was fixed in 4% paraformaldehyde for 24 h and subsequently embedded in paraffin. Paraffin-embedded heart tissue was sectioned into 5 μm-thick slices, which were then dewaxed. Hematoxylin and eosin staining were performed for 5 min each on the sections. After staining, the slices were observed under a microscope and images were captured.

### TTC staining

After collection, hearts were washed and frozen at −80°C for 10 min and then sliced into 2 mm thick sections, which were subsequently incubated in 2,3,5-triphenyltetrazolium chloride (TTC) solution in the dark at 37°C for 20 min. Following incubation, the tissue slices were fixed in 4% paraformaldehyde and images of the tissue were photographed. The myocardial infarct size and viable tissue area were calculated using Image Pro Plus 6.0.

### Transmission Electron Microscopy

Fresh heart tissues were cut into 1 mm^3^ blocks and then fixed with an electron microscope fixative and 1% osmic acid. The fixed heart tissues were subsequently dehydrated and embedded, and cut into 60–80 nm slices. The slices were then stained sequentially with a 2% uranium acetate saturated alcohol solution and a 2.6% lead citrate solution. Following staining, the slices were observed under a transmission electron microscope (TEM).

### Western blot

Total protein was extracted from heart or H9C2 using RIPA lysis buffer (R0010, Servicebio, China). The concentration of total protein was quantified by BCA protein assay kit (PC0020, Servicebio, China). Samples were then separated by 10% sodium dodecyl sulfate polyacrylamide gel electrophoresis (SDS-PAGE) and transferred to 0.45 μm polyvinylidene difluoride (PVDF) membranes (Millipore, United States). Following transfer, PVDF membranes were blocked by 5% skim milk and incubated with primary antibodies including TFR1 (ab214039, abcam, United Kingdom), GPX4 (ab125066, abcam, United Kingdom), GAPDH (60004-1-Ig, Proteintech, China), Sat1 (10708-1-AP, Proteintech, China), P-ERK (4,370, CST, United States), ERK (11257-1-AP, Proteintech, China), P-JNK (4,668, CST, United States), JNK (9,252, CST, United States), P-P38 (4,511, CST, United States), P38 (9,218, CST, United States). After incubation overnight, PVDF membranes were incubated with HRP-conjugated affinipure goat anti-mouse/rabbit IgG (SA00001-1/SA00001-2, Proteintech, China) for 1 h at room temperature. Finally, the protein blot was visualized by Tanon imaging system (Tanon-5200, China) and quantified using ImageJ software.

### Cell viability assays

The 96-well plates were seeded with H9C2 and incubated for 24 h. Next, after normal incubation for three and a half hours or OGD/R, 10 μL of Cell Counting Kit-8 (CCK-8) solution (K1018, Apexbio, United States) was added to each well and incubated for 2.5 h. The absorbance value at 450 nm was measured using a microplate reader.

### Measurement of LDH activity

Lactate Dehydrogenase (LDH) activity was measured by a standard kit (A020-2, Nanjing Jiancheng Bioengineering, China). Rat serum or cell supernatant was collected and mixed with the reaction solution according to the manufacturer’s instructions. After 30 min of incubation at 37°C, the absorbance value at 450 nm was measured using a microplate reader.

### Measurement of GSH and LPO

The levels of glutathione (GSH) and lipid peroxidation (LPO) in plasma and H9C2 cells were measured using standard kits (A006-2-1 for GSH, and A106-1-2 for LPO; Nanjing Jiancheng Bioengineering, China) according to manufacturer’s instructions. The absorbance of the samples was measured at 405 nm (GSH) and 586 nm (LPO) using a microplate reader.

### Measurement of cellular lipid peroxidation

Cellular lipid peroxidation was measured using a lipid peroxidation sensor (D3861, Thermo, United States) according to manufacturer’s instruction. After inducing OGD/R in H9C2 cells, a 5 µM lipid peroxidation probe was added to the culture dish and incubated, and the cells were incubated for 30 min. The cells were then washed three times with cold PBS, and the cell pellets were collected and analyzed for fluorescence intensity using a Cytoflex (Beckman Coulter, United States). The fluorescence data were analyzed using Flowjo V10.

### Tissue ROS staining

To measure the ROS level of cardiac tissue, heart was embedded with optimal cutting temperature compound and placed in the refrigerator at −80°C, the frozen heart was cut into 6 μm slices and washed them with cold PBS. Next, slices were incubated with 2.5 μmol DCFH-DA at 37°C for 30 min and washed with PBS. Lastly, slices were incubated with DAPI for 8 min, then the slices were washed, fixed and photographed.

### RNA extraction and real-time quantitative polymerase chain reaction (qPCR)

Total RNA was extracted from H9C2 or myocardial tissue using RNAzol reagent (RN190, Molecular Research Center Inc., United States) according to the manufacturer’s instructions. A total of 800 ng RNA was used to synthesize cDNA using ABScript III RT Master Mix (RK20429, Abclonal, China). PCR amplification of cDNA was performed using Genious 2X SYBR Green Fast qPCR Mix (RK21205, Abclonal, China) following the manufacturer’s instructions. Relative mRNA expression levels were normalized to β -actin and analyzed using the 2^−ΔΔCT^ method. The primers used in this study were designed by Sangon Biotech (Shanghai, China): Sat1-Forward: AAC​GAA​TGA​GGA​ACC​ACC​T; Sat1-Reverse: GTGGCTGGACGGATCTT. β-actin-Forward: CCC​ATC​TAT​GAG​GGT​TAC​GC; β-actin-Reverse: TTT​AAT​GTC​ACG​CA-CGA​TTT​C.

### Differentially expressed genes analysis and download of ferroptosis-related genes

The GEO dataset (https://www.ncbi.nlm.nih.gov/gds/) was used to retrieve relevant datasets about MIRI. Using a threshold of |log fold change (FC)| > 0.589 (FC > 1.5) and *P* < 0.05, the differentially expressed genes (DEGs) were analyzed using the R package Linear Models for Microarray Data (LIMMA). The expression pattern of the DEGs was presented using volcano plots. FerdbV2 database was used to download ferroptosis-related genes (Zhou et al., 2023).

### Construction of gene-interaction network and hub gene identification

Online webtool Comparative Toxicogenomics Database (CTD, http://ctdbase.org/) was used to search genes that interacted with Sat1. Then the gene interactions were imported into Cytoscape (https://cytoscape.org) for visualization of the gene-interaction network. Subsequently, the top 10 hub genes with the highest connectivity score were extracted using the CytoHubba plugin.

### Function and pathway analysis

Gene Ontology (GO) database was utilized to perform functional analysis of Sat1 and its interactional genes in biological process (BP), cellular component (CC), and molecular function (MF) categories by using the R package clusterProfiler. Pathway analysis was conducted using the Kyoto Encyclopedia of Genes and Genomes (KEGG) database, and the signaling pathways of DEGs were investigated using the R package clusterProfiler.

### Transcription factor analysis

Potential transcription factors can be queried by entering the FASTA sequence of Sat1 into the TFDB, ALYGGEN and ChEA3 databases. The query method for JASPAR database is as follows: the potential promoter region base sequence of Sat1 was obtained by search of the NCBI Gene database (https://www.ncbi.nlm.nih.gov/gene/). The transcription factors of Sat1 were obtained through JASPAR hubs of the UCSC database (http://genome.ucsc.edu/). Transcription factors showed same direction and with Sat1 and had a score over 500 were considered as potential transcription factors (Shahrajabian and Sun, 2023).

### Luciferase reporter gene experiment

After transfecting the plasmid for 36 h, the cells were cleaned 3 times with PBS, 500 μL special cell lysate was added to the 6-well plate, and incubated on ice for 5 min to fully lysate the cells. Then the cell suspension was collected and centrifuged at 12,000 rpm for 1 min. Then, 20 μL cell suspension was added into the 96-well plate, and 100 μL firefly luciferase detection reagent was added, and the fluorescence intensity was detected by multimode reader.

### Statistical analysis

The results are presented as mean ± standard deviation. Statistical analysis was performed using GraphPad Prism 6.0. Differences between the two groups were evaluated using the Student’s t-test, while One-way ANOVA was used for comparisons among multiple groups, and Fisher’s LSD test was used for comparisons between two groups. *P* value <0.05 was considered statistically significant.

## Results

### Ferroptosis was involved in a rat model of MIRI

To demonstrate the induction of ferroptosis in MIRI, a rat model of MIRI was established. The results demonstrate a notable elevation in the ST wave of the MIRI group (Supplementary Figure S1A–B). Furthermore, cardiac fiber loss and cardiomyocyte disarrangement were increased in MIRI (Figure 1A). Additionally, TTC staining showed an increase in the infarct size in MIRI (Figures 1B, C). To explore the role of ferroptosis in MIRI, changes in GSH, LPO, and ROS levels were investigated as they are important indicators of ferroptosis activation. The data presented in Figures 1D, E showed that serum levels of GSH and LPO were significantly decreased and increased in MIRI, respectively. Moreover, an increase in ROS accumulation was observed in the myocardium of the MIRI group (Figure 1F). The results obtained from TEM analysis indicated a significant increase in disorganized mitochondrial cristae and mitochondrial membrane disruption in MIRI (Figure 1G). An increase in Transferrin Receptor 1 (TFR1) and a decrease in GPX4 protein expression were also detected in MIRI, which are key markers in the ferroptosis signaling pathway (Figures 1H–J). These results collectively indicate that ferroptosis may play a role in the MIRI model.

### Identification of ferroptosis-related genes and pathways in MIRI

**FIGURE 2:**
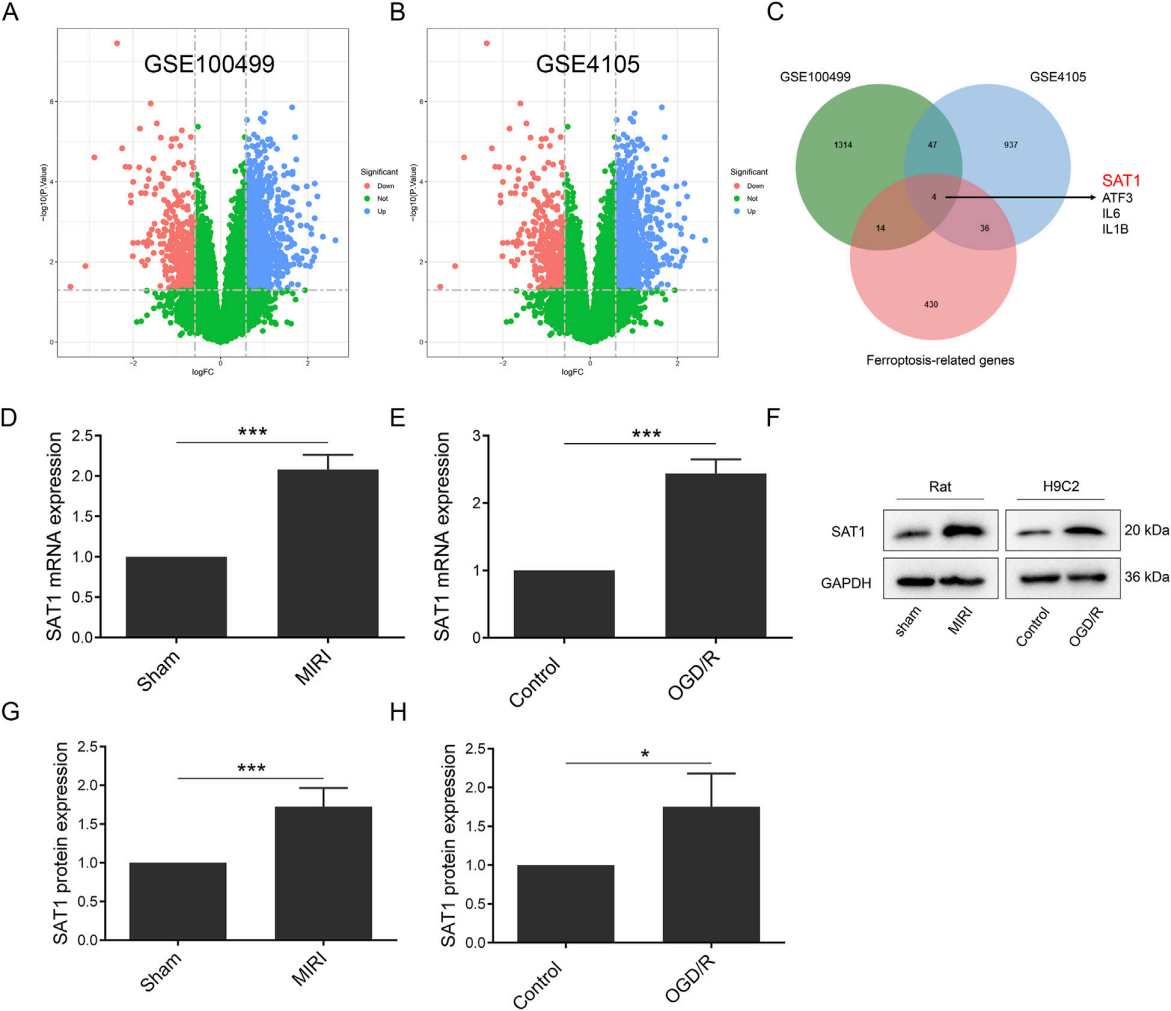
Sat1 was identified as ferroptosis-related DEGs. **(A)** Volcano map of DEGs in GSE100499; **(B)** Volcano map of DEGs in GSE4105; **(C)** Venn diagram of DEGs in GSE100499 and GSE4105 and ferroptosis-related genes; **(D)** Sat1 mRNA expression in rat; **(E)** Sat1 mRNA expression in H9C2; **(F)** Protein expression of SAT1 in rat and H9C2; **(G)** Quantification of relative protein expression level of SAT1 in rat; **(H)** Quantification of relative protein expression level of SAT1 in H9C2. (n = 4 for each group). Significance levels are denoted by *P < 0.05; ***P < 0.001.

For the identification of ferroptosis-related genes involved in MIRI, we searched the GEO dataset and analyzed the differentially expressed genes (DEGs) in GSE100499 and GSE4105. In GSE100499, there were 942 upregulated and 437 downregulated genes, and there were 334 upregulated and 690 downregulated genes in GSE4105. The expression pattern of the DEGs was displayed in a volcano plot (Figures 2A, B). After intersecting the DEGs with ferroptosis-related genes, SAT1, ATF3, IL6 and IL1B were identified as ferroptosis-related DEGs (Figure 2C). Through literature search, SAT1 was selected as potential target to study. It was found that the mRNA and protein levels of SAT1 were both significantly increased in rat MIRI model and H9C2 OGD/R model (Figures 2D–H).

### Inhibition of Sat1 alleviates MIRI by regulating ferroptosis *in vivo*

**FIGURE 3:**
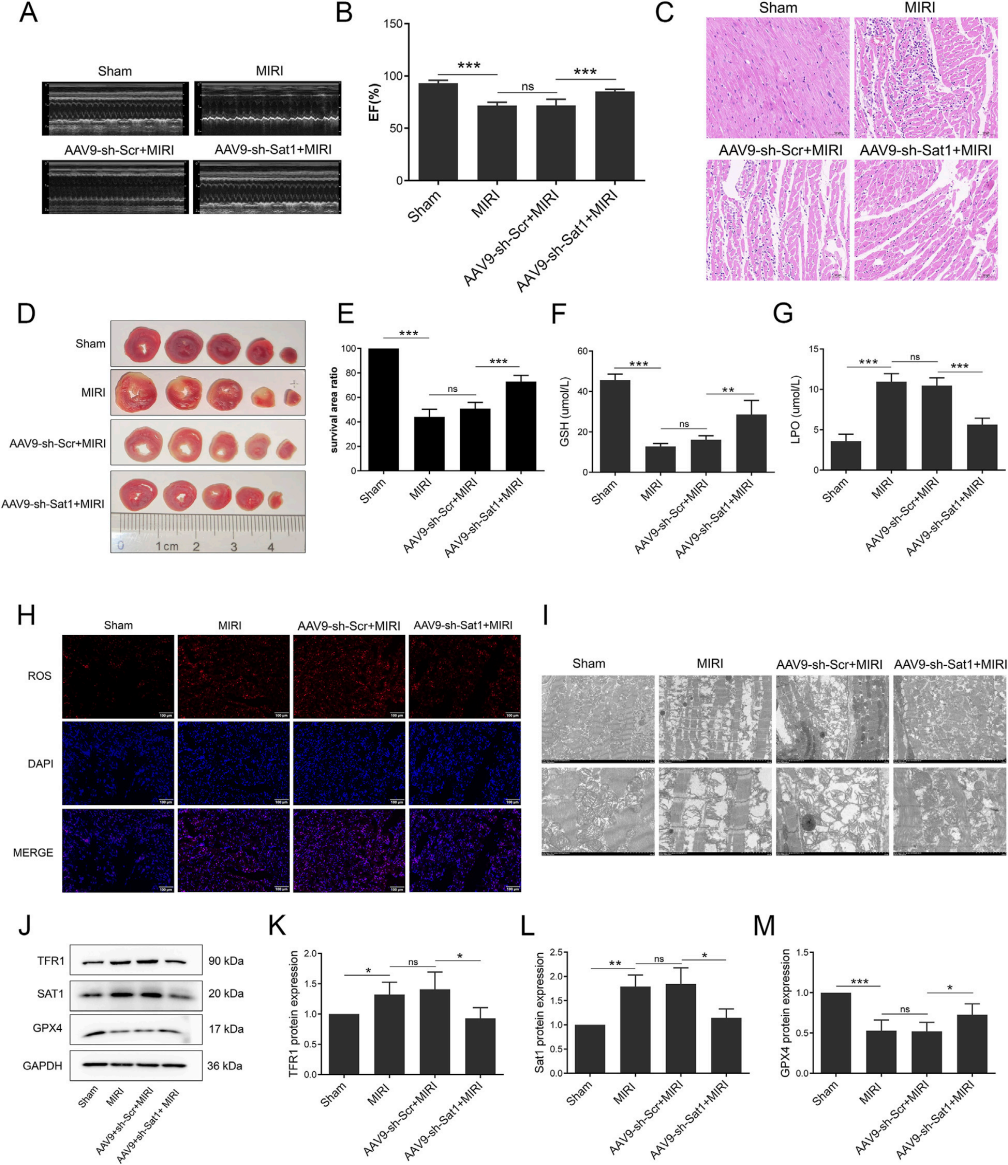
Inhibition of Sat1 alleviated MIRI via regulation of ferroptosis *in vivo*. **(A)** M-mode echocardiography of rat heart; **(B)** Measurement of ejection fraction; **(C)** HE staining of heart tissue; **(D)** TTC staining of heart tissue; **(E)** Quantification of infarct size; **(F)** Serum level of GSH; **(G)** Serum level of LPO; **(H)** ROS staining of heart tissue; **(I)** Mitochondria morphology of heart tissue; **(J)** Protein expression of TFR1, SAT1, GPX4 and GAPDH in rat; **(K–M)** Quantification of relative protein expression level of TFR1, SAT1 and GPX4 (n = 4 for each group). Significance levels are denoted by *P < 0.05; **P < 0.01; ***P < 0.001.

To further investigate the role of Sat1 in MIRI *in vivo*, AAV9 was applied in SD rats to suppress Sat1 expression. As shown in Figure 3, Sat1 inhibition significantly increased EF (Figures 3A, B), ameliorated cardiac damage (Figure 3C) and reduced myocardial infarction size (Figures 3D, E). Since the protective effect of Sat1 inhibition was demonstrated *in vivo*, we then explored its effect on ferroptosis. The results showed that, compared to AAV9-sh-Scr + MIRI group, GSH was increased and LPO was decreased in AAV9-sh-Sat1+MIRI group (Figures 3F, G). Morphologically, suppressing Sat1 expression significantly decreased ROS accumulation (Figure 3H) and mitochondrial damage (Figure 3I). Moreover, after inhibition of Sat1, compared to AAV9-sh-Scr + MIRI group, increased TFR1 protein expression and decreased GPX4 protein expression were obviously reversed in AAV9-sh-Sat1+MIRI group (Figures 3J–M). Therefore, these results above demonstrated that inhibition of Sat1 can alleviate MIRI by regulation of ferroptosis *in vivo*.

### Inhibition of Sat1 alleviates OGD/R by regulating ferroptosis *in vitro*

**FIGURE 4:**
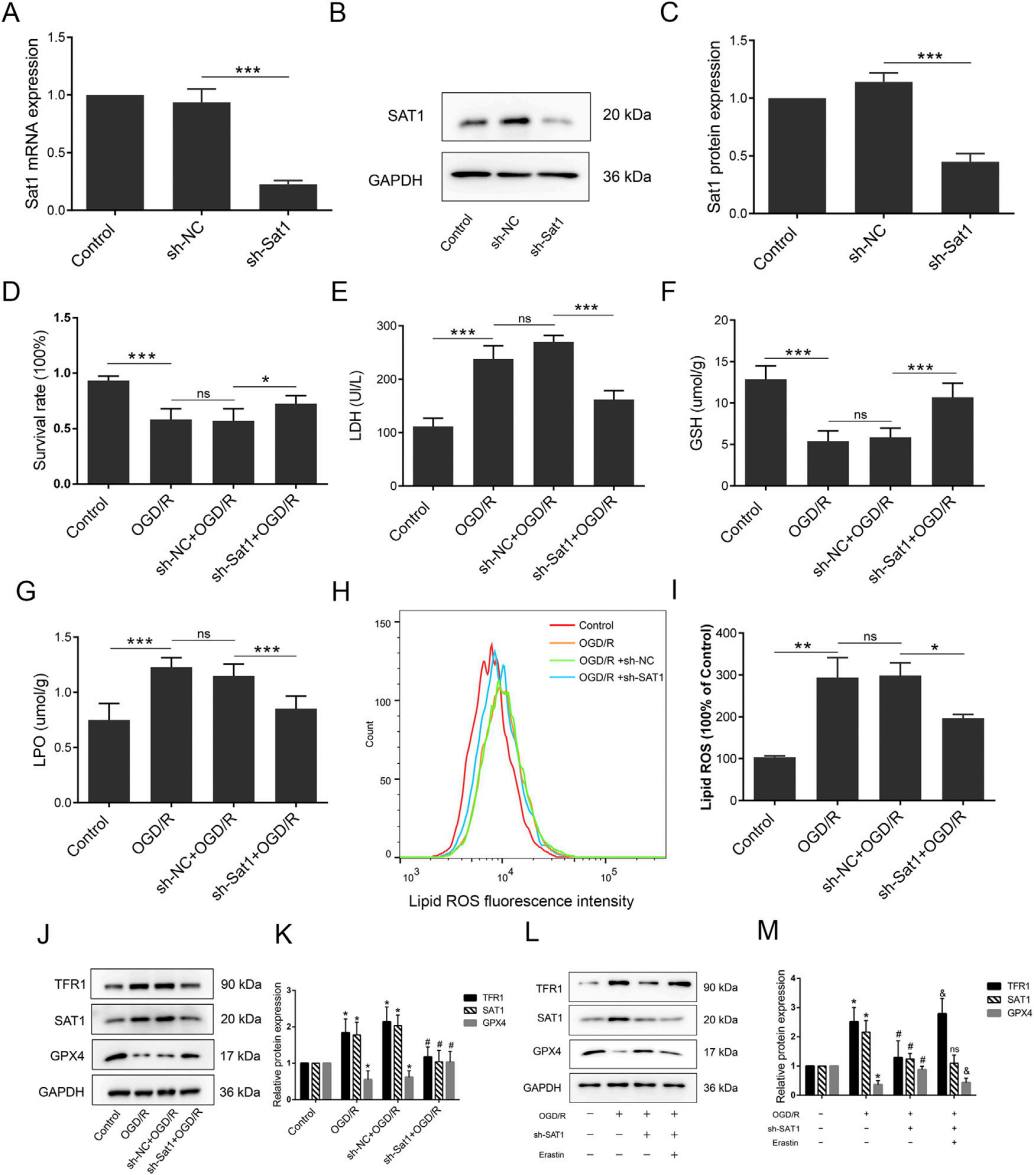
Inhibition of Sat1 alleviated MIRI via regulation of ferroptosis *in vitro*. H9C2 were treated with OGD/R with or without Sat1 inhibition. **(A)** Sat1 mRNA expression in H9C2; **(B)** Protein expression of SAT1 in H9C2; **(C)** Quantification of relative protein expression level of SAT1; **(D)** Cell viability was detected by CCK8; **(E)** Cellular LDH release; **(F)** Cellular GSH content; **(G)** Cellular LPO content; **(H)** Cellular ROS was detected by lipid peroxidation sensor; **(I)** Quantification of ROS fluorescence intensity; **(J, L)** Protein expression of TFR1, SAT1, GPX4 and GAPDH in H9C2; **(K, L)** Quantification of the relative protein expression level of TFR1, SAT1, and GPX4 (n = 4 for each group). For Figure **(A–I)**, significance levels are denoted by *P < 0.05; **P < 0.01; ***P < 0.001. For Figure **(K, M)**, *OGD/R vs. Control, *P* < 0.05; ^#^sh-NC vs. sh-Sat1, *P* < 0.05; ^&^sh- Sat1 vs. Erastin, *P* < 0.05; ^ns^sh-NC vs. sh-Sat1, difference was not significant.

To study the role of Sat1 in OGD/R *in vitro*, lentiviral transduction was performed to suppress Sat1 expression and observe the effect on ferroptosis and MIRI. After successfully knockdown of Sat1 mRNA and protein expression in H9C2 (Figures 4A–C), it was observed that the inhibition of Sat1 significantly rescued cell viability (Figure 4D) and decreased LDH release after OGD/R (Figure 4E). In order to investigate the effect of Sat1 inhibition on ferroptosis, the GSH level and the level of LPO were analyzed. The results showed that the GSH level was restored, and the level of LPO was significantly decreased in the sh-Sat1+OGD/R group compared to the sh-NC + OGD/R group (Figures 4F, G). Sat1 inhibition resulted in the reversal of increased lipid peroxidation levels, as shown by the results of flow cytometry (Figures 4H, I). Moreover, increased TFR1 protein expression and decreased GPX4 protein expression were both significantly reversed in the sh-Sat1+OGD/R group compared to the sh-NC + OGD/R group (Figures 4J, K). Moreover, combination of Sat1 knockdown and ferroptosis activator erastin was performed to clarify the relationship between Sat1 and ferroptosis. As shown in Figures 4L, M, erastin significantly reversed the effect of Sat1 knockdown on TFR1 and GPX4 protein expression, but it did not affect the protein expression of SAT1, which revealed that SAT1 was the upstream regulator of ferroptosis. These results collectively indicated that inhibition of Sat1 alleviated OGD/R via regulation of ferroptosis *in vitro*.

### Over-expression of Sat1 aggravates OGD/R by regulating ferroptosis *in vitro*

**FIGURE 5:**
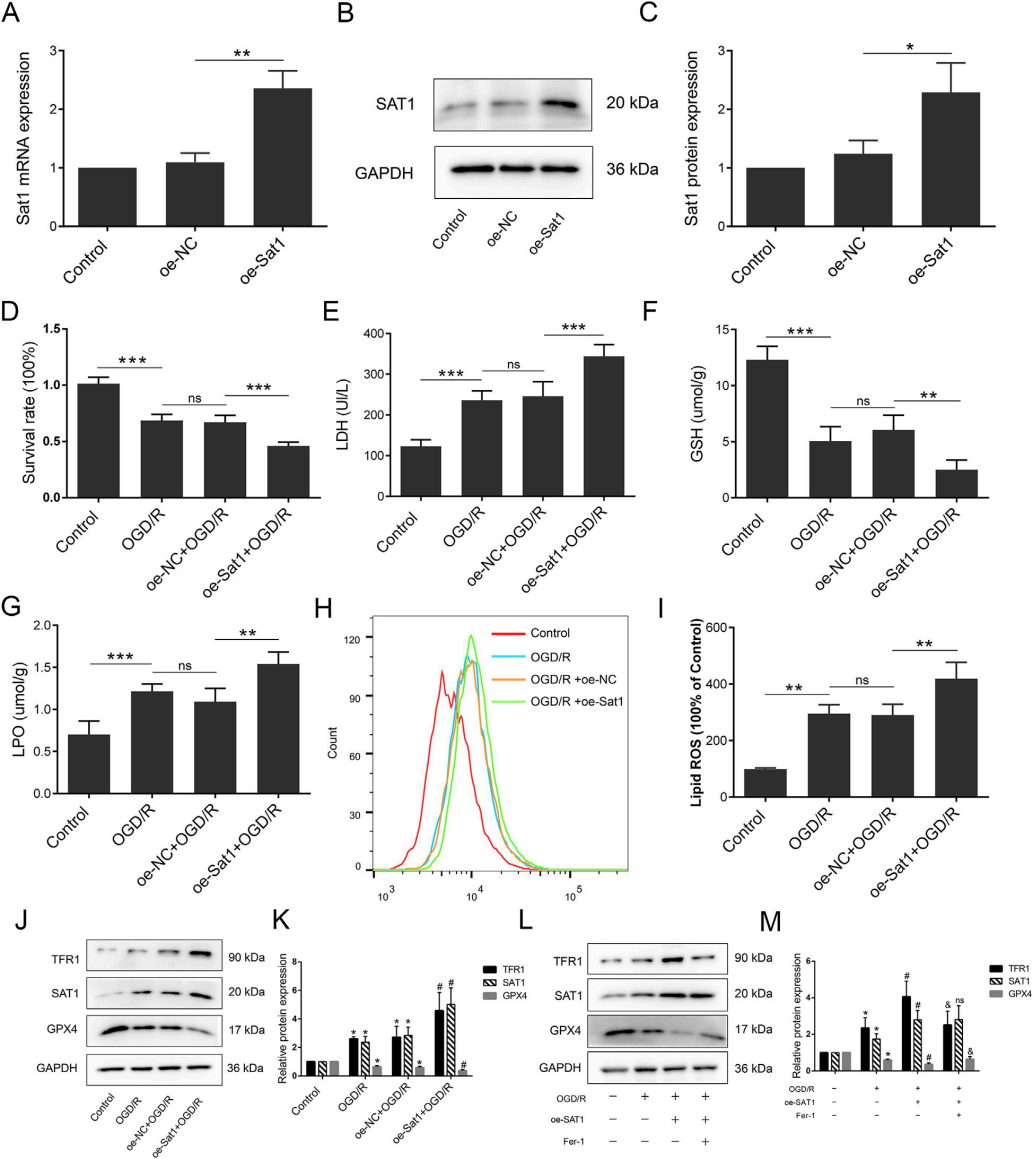
Over-expression of Sat1 aggravated MIRI through ferroptosis. H9C2 were treated with OGD/R with or without Sat1 over-expression. **(A)** Sat1 mRNA expression in H9C2; **(B)** Protein expression of SAT1 in H9C2; **(C)** Quantification of relative protein expression level of SAT1; **(D)** Cell viability was detected by CCK8; **(E)** Cellular LDH release; **(F)** Cellular GSH content; **(G)** Cellular LPO content; **(H)** Cellular ROS was detected by lipid peroxidation sensor; **(I)** Quantification of ROS fluorescence intensity; **(J, L)** Protein expression of TFR1, SAT1, GPX4 and GAPDH in H9C2; **(K, M)** Quantification of the relative protein expression level of TFR1, SAT1, and GPX4 (n = 4 for each group). For Figure **(A–I)**, significance levels are denoted by *P < 0.05; **P < 0.01; ***P < 0.001. For Figure **(K, M)**, *OGD/R vs. Control, *P* < 0.05; ^#^oe-NC vs. oe-Sat1, *P* < 0.05; ^&^oe- Sat1 vs. Fer-1, *P* < 0.05. ^ns^oe-NC vs. oe-Sat1, difference was not significant.

After confirming the effect of Sat1 inhibition on MIRI, we explored whether over-expression of Sat1 promotes OGD/R via ferroptosis regulation. After successfully over-expressing Sat1 mRNA and protein expression in H9C2 (Figures 5A–C), the results showed that over-expression of Sat1 significantly inhibited cell viability (Figure 5D) and increased LDH release after OGD/R (Figure 5E). The GSH level was decreased, and the level of LPO was significantly increased in the oe-Sat1+OGD/R group compared to the oe-NC + OGD/R group (Figures 5F, G). The results of flow cytometry showed that Sat1 over-expression resulted in increased lipid peroxidation levels (Figures 5H, I). Moreover, Sat1 over-expression further promoted TFR1 protein expression and decreased GPX4 protein expression (Figures 5J, K). Moreover, combination of Sat1 over-expression and ferroptosis suppressor Fer-1 was performed in H9C2 to explore the relationship between Sat1 and ferroptosis. As shown in Figures 5L, M, Fer-1 significantly reversed the effect of Sat1 over-expression on TFR1 and GPX4 protein expression, but it did not affect the protein expression of SAT1, which also demonstrated that SAT1 was the upstream regulator of ferroptosis. These results jointly revealed that over-expression of Sat1 aggravated OGD/R through regulation of ferroptosis.

### Functional and pathway analysis of SAT1-Interacting genes

**FIGURE 6:**
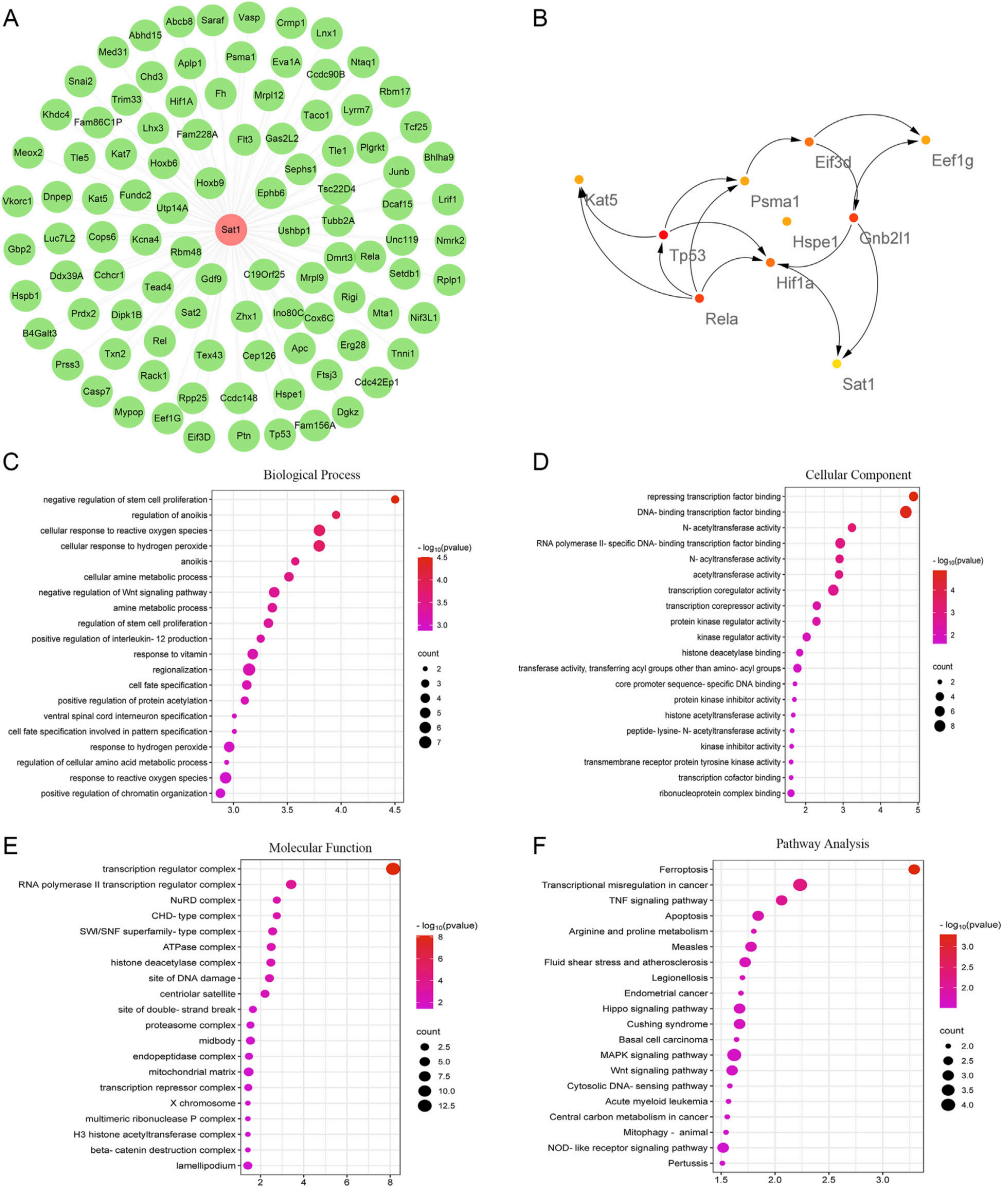
Interactional genes of Sat1 and functional and pathway analysis. **(A)** Network of Sat1 and its interactional genes; **(B)** The top 10 hub genes among Sat1 and its interactional genes; **(C)** BP of Sat1 and its interactional genes; **(D)** CC of Sat1 and its interactional genes; **(E)** MF of Sat1 and its interactional genes; **(F)** Signaling pathways of Sat1 and its interactional genes.

After confirming the role of Sat1 in MIRI, to find the pathway involved in the regulation of Sat1 to MIRI, the CTD database was searched to identify genes that interact with Sat1, and their functions and signaling pathways were analyzed. The gene-interaction network analysis showed that Sat1 interacted with 99 genes, including Sat2, Hoxb9, and Rbm48 (Figure 6A). Subsequently, these genes were further analyzed in the STRING database to identify essential genes. It was found that among the identified genes, Kat5, Tp53, Rela, Hif1a, Hspe1, Psma1, Sat1, Eif3d, Gnb2l1, and Eef1g were the top 10 hub genes (Figure 6B). Next, GO and KEGG analyses were performed to uncover the biological functions and signaling pathways of these interactional genes. GO analysis revealed that Sat1 and its interactional genes are mainly enriched in the BP of “negative regulation of stem cell proliferation”, “regulation of anoikis”, and “cellular response to reactive oxygen species” (Figure 6C). The CC of Sat1 and its interactional genes are mainly enriched in “transcription regulator complex”, “RNA polymerase II transcription regulator complex”, and “NuRD complex” (Figure 6D). Sat1 and its interactional genes mainly participated in MF of “Repressing transcription factor binding”, “DNA-binding transcription factor binding”, and “N-acetyltransferase activity” (Figure 6E). In addition, KEGG pathway analysis indicated that Sat1 and its interactional genes are mainly enriched in “Ferroptosis”, “TNF signaling pathway”, “Apoptosis”, “MAPK signaling pathway”, and “Wnt signaling pathway” (Figure 6F).

### Sat1 affects MIRI by regulation of ferroptosis through MAPK/ERK pathway

**FIGURE 7:**
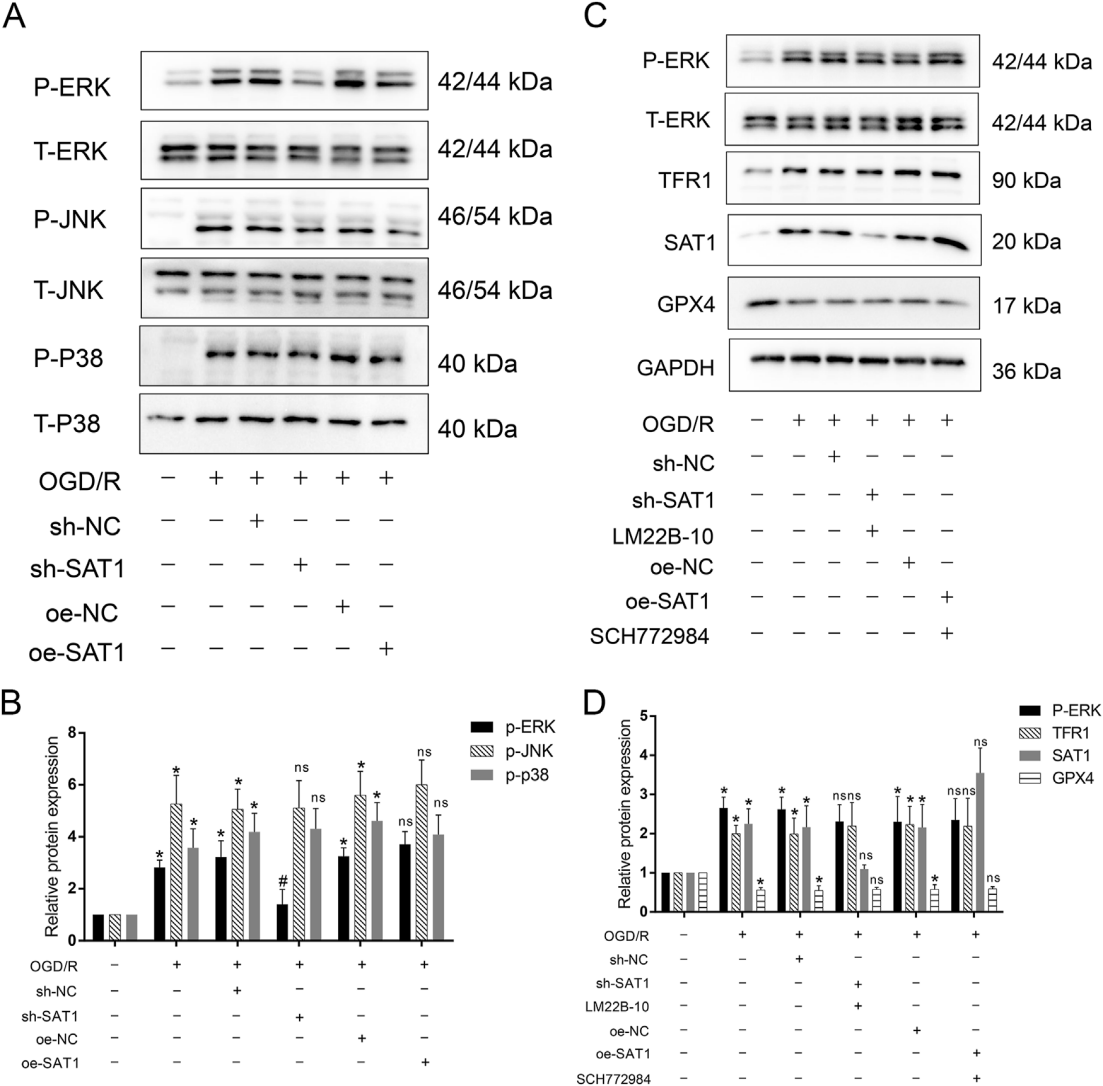
The effect of Sat1 expression on MAPK pathway activation. **(A)** Protein expression of P-ERK, T-ERK, P-JNK, T-JNK and P-P38, T-P38; **(B)** Relative protein expression of P-ERK/T-ERK, P-JNK/T-JNK; P-P38/T-P38; **(C)** Protein expression of P-ERK, ERK, TFR1, SAT1 and GPX4; **(D)** Relative protein expression of P-ERK/T-ERK, TFR1, SAT1 and GPX4 (n = 4 for each group). Significance levels are denoted by *OGD/R vs. Control, *P* < 0.05; ^#^sh-NC vs. sh-Sat1, *P* < 0.05; ^ns^sh-NC vs. sh-Sat1 or ^ns^oe-NC vs. oe-Sat1, difference was not significant.

As results showed above, bioinformatic analysis indicated the MAPK pathway which Sat1 may regulate. Since MAPK pathway played important role in MIRI, we tested the activation of MAPK pathway, including ERK, JNK and P38. The results showed that ERK, JNK and P38 were all activated after MIRI (Figures 7A, B), among them, Sat1 inhibition only significantly suppressed the activation of MAPK/ERK, but Sat1 over-expression did not significantly promote the activation of MAPK/ERK (Figure 7B). Then, rescue experiments were performed to further demonstrate the relationship between Sat1 and MAPK/ERK. The results showed that, in sh-SAT1 group, after using ERK pathway activator of LM22B-10, phosphorylated-ERK was increased (Figures 7C, D). Increased phosphorylation of ERK reversed the effect of Sat1 inhibition on TFR1 and GPX4 expression, but it has no effect on expression of Sat1 (Figures 7C, D). Moreover, in oe-SAT1 group, after using MAPK/ERK pathway suppresser of SCH772984, phosphorylated-ERK was decreased (Figures 7A, B). Decreased phosphorylation of ERK significantly suppressed TFR1 expression and increased GPX4 expression, but it also did not affect the expression of Sat1 (Figures 7C, D). These results identified MAPK/ERK pathway as downstream target of Sat1, and MAPK/ERK pathway can affect the activation of ferroptosis, which demonstrated that Sat1 play important role in MIRI through regulation of ferroptosis via MAPK/ERK pathway.

### SOX2 transcriptionally suppress Sat1 expression

**FIGURE 8:**
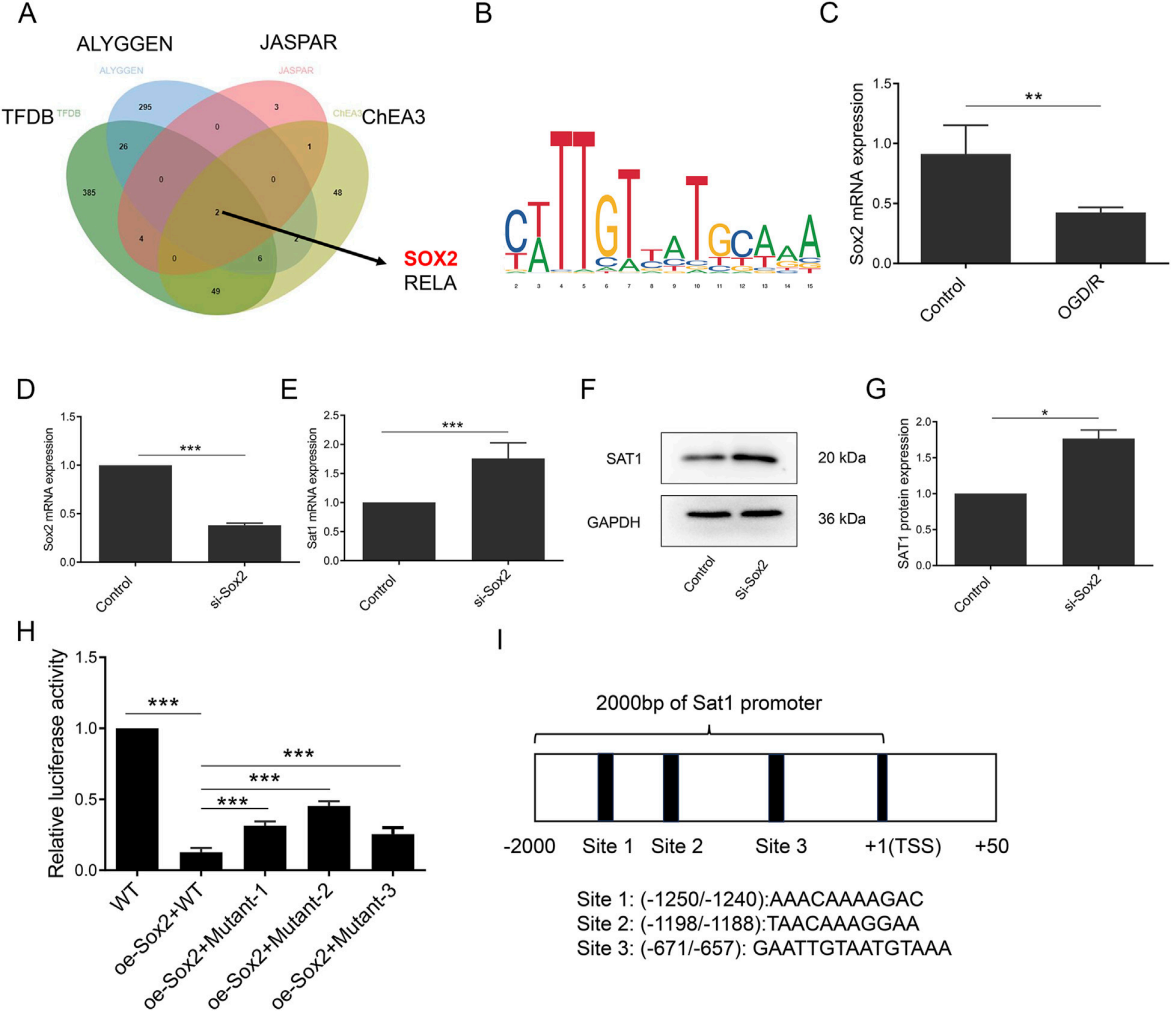
Sox2 was identified transcription factor of Sat1. **(A)** Predication of transcription factor of Sat1 by databases of TFDB, ALYGGEN, JASPAR and ChEA3; **(B)** Potential sites of Sox2 binding to Sat1; **(C, D)** Sox2 mRNA expression; **(E)** Sat1 mRNA expression; **(F)** SAT1 and GAPDH protein expression; **(G)** Quantification of the relative protein expression level of SAT1; **(H)** Relative luciferase activity of each group; **(I)** schematic diagram of plasmids with mutant sequence of Sat1 (n = 4 for each group). Significance levels are denoted by *P < 0.05; **P < 0.01; ***P < 0.001.

As shown in Figure 6, bioinformatic analysis showed that Sat1 and its interactional genes were enriched in CC of “transcription regulator complex” and “RNA polymerase II transcription regulator complex”, (Figure 6D). Sat1 and its interactional genes mainly participated in MF of “Repressing transcription factor binding” and “DNA-binding transcription factor binding” (Figure 6E), which revealed that regulation at the transcription level may be associated with the abnormal Sat1 expression in MIRI. Therefore, we predicted the transcription factor of Sat1 through search of four databases, including TFDB, ALYGGEN, JASPAR and ChEA3. It was found that SOX2 and RELA may be the transcription factor of Sat1 (Figure 8A). Through literature retrieval, we excluded RELA and select SOX2 to study it whether transcriptionally regulate Sat1 expression. TFDB database was used to predict the potential binding site of SOX2 to Sat1 (Figure 8B). In OGD/R, Sox2 mRNA expression was significantly decreased (Figure 8C). Next, we construct siRNA to suppress Sox2 expression in normal H9C2. After successfully suppression of Sox2 expression, it is revealed that Sat1 mRNA and protein expression were significantly increased (Figures 8D–G), which demonstrated that Sox2 suppressed Sat1 expression in normal condition. Then, plasmids of wild type Sat1, mutant Sat1 and over-expression of Sox2 were constructed. After different combinations of plasmids were transfected into H9C2, it was observed that compared with WT group, relative luciferase activity was significantly decreased in oe-Sox2 + WT group (Figure 8H). Compared with oe-Sox2+WT group, relative luciferase activity was all increased in oe-Sox2+Mutant1/2/3 group, and the increase was most obvious in oe-Sox2+Mutant2 group (Figure 8H), indicating that the Mutant Site 2 (TAACAAAGGAA) maybe the potential binding site of Sox2 regulating Sat1(Figure 8I). These results collectively demonstrated that SOX2 suppress Sat1 expression at the transcription level.

## Discussion

Myocardial ischemia-reperfusion injury, commonly observed in patients with myocardial ischemia, remains a challenging issue that requires urgent resolution. Accumulating literature has indicated the crucial role of ferroptosis in the development of MIRI (Liu et al., 2021; Chen et al., 2021; Lu et al., 2022). Therefore, targeting ferroptosis as a therapeutic strategy could be a promising approach to developing treatments for MIRI.

**FIGURE 9:**
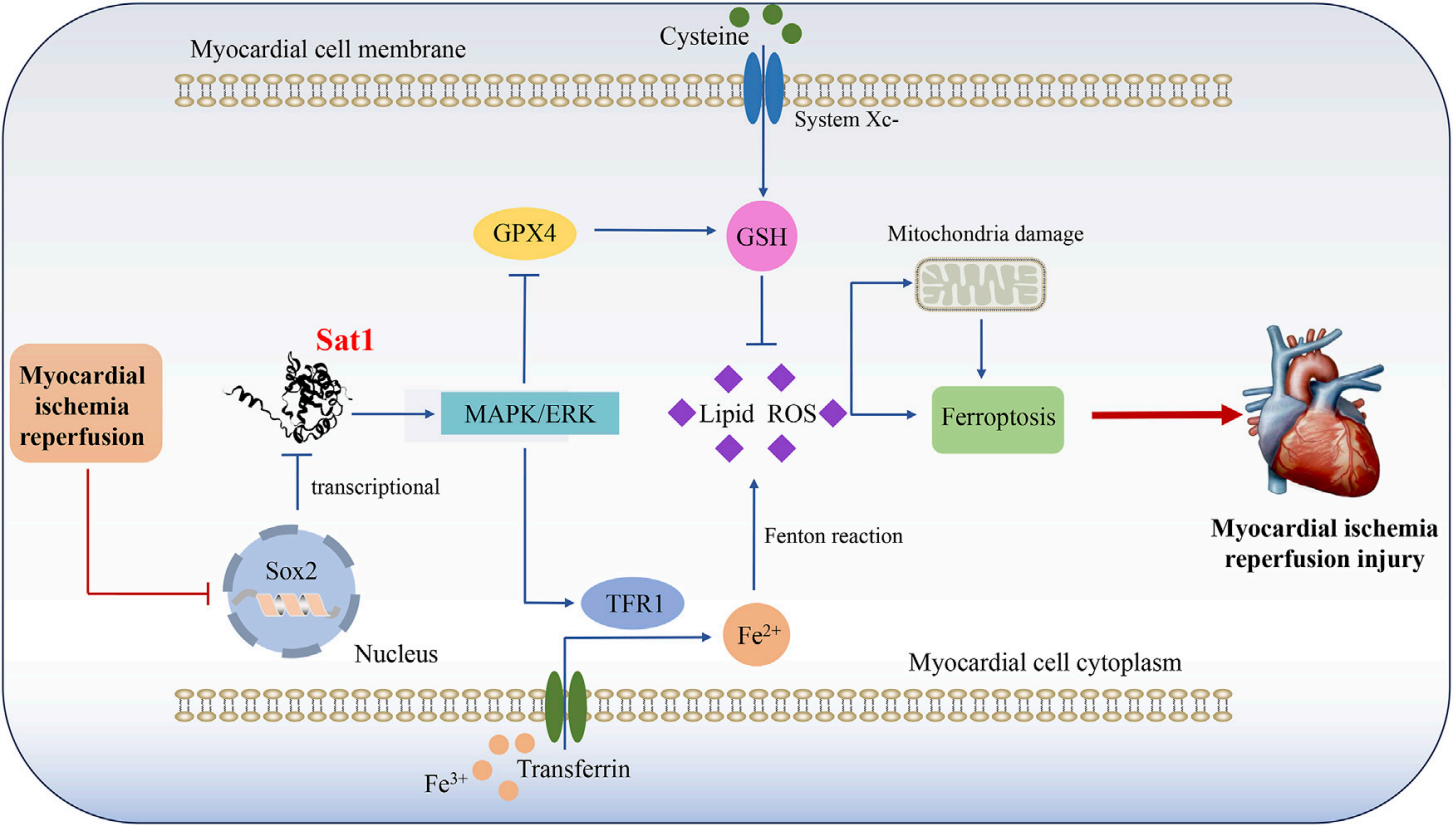
Mechanism diagram shows that Sat1, which transcriptionally suppressed by Sox2, participate in the development of MIRI by regulation of ferroptosis through MAPK/ERK pathway.

This study demonstrated successful induction of ferroptosis in MIRI. Importantly, bioinformatic analysis, q-PCR, and western blot provided evidence that Sat1 was upregulated in MIRI. Sat1 and its interactional genes were enriched in various biological processes and pathways, including ferroptosis and the MAPK signaling pathway. Furthermore, the inhibition of Sat1 was shown to alleviate MIRI by regulating ferroptosis *in vitro* and *in vivo*, and over-expression of Sat1 aggravated MIRI through the regulation of ferroptosis. Importantly, Sat1 can regulate the activation of MAPK/ERK pathway, then affect MIRI through regulation of ferroptosis. Moreover, SOX2 suppress Sat1 expression at the transcription level (Figure 9).

Iron homeostasis is vital for maintaining normal cellular functions. However, excessive iron accumulation can lead to ferroptosis, which is morphologically, biochemically, and genetically distinct from other forms of cell death, including pyroptosis, apoptosis, and autophagy (Fang et al., 2019). Growing evidence has demonstrated that ferroptosis plays a significant role in multiple pathological processes, including MIRI (Miyamoto et al., 2022; Sun et al., 2022; Yang et al., 2023). Studies have reported that increased iron load, accumulation of ROS, lipid peroxidation, and disturbance of the anti-oxidation system are all related to the occurrence of MIRI (He et al., 2022; Ying et al., 2022). Dexmedetomidine has been shown to decrease oxidative stress, Fe^2+^ accumulation, lipid peroxidation, and restore the expression GPX4, ultimately protecting against MIRI via regulating ferroptosis (Ma et al., 2023; Wang et al., 2022; Yu et al., 2022). Our findings revealed significant changes in mitochondrial morphology in MIRI, accompanied by increased LPO content, ROS accumulation, TFR1 protein expression, and decreased GSH content and GPX4 protein expression. These results collectively demonstrate the crucial role of ferroptosis in MIRI and highlight the significance of identifying potential biomarkers for the treatment of MIRI.

Accordingly, we performed bioinformatics analysis to identify the DEGs related to ferroptosis. Notably, Sat1 was identified as potential target and it was significantly increased in MIRI at the mRNA and protein levels. Related studies have also highlighted the importance of Sat1 in ferroptosis. A recent study has shown that the activation of Sat1 can lead to lipid peroxidation and ferroptosis in response to ROS intervention via regulation of ALOX15 (Ou et al., 2016). Increased expression of Sat1 at the mRNA and protein levels has been associated with immune cell infiltration and poor outcomes for patients with low-grade glioma (Mou et al., 2022). However, the specific mechanism by which Sat1 regulates MIRI needs further investigation. Hence, the interactional genes of Sat1 were identified, and functional and pathway analyses were conducted using GO and KEGG. The results revealed that 99 genes interacted with Sat1 and were enriched in the cellular response to ROS, the TNF signaling pathway, negative regulation of the Wnt signaling pathway, repressing transcription factor binding, ferroptosis, and the MAPK signaling pathway. Excessive production of ROS is a crucial factor in the development of MIRI (Chouchani et al., 2014). Moreover, ROS accumulation is also considered as a primary contributory factor to ferroptosis, which is characterized by iron-dependent lipid peroxidation. During MIRI, the accumulation of irons leads to the excessive generation of ROS, resulting in lipid peroxidation and myocardial injury. These findings indicate that Sat1 may participate in the regulation of MIRI through ferroptosis. To investigate Sat1 whether regulates MIRI via ferroptosis, Sat1 expression was suppressed *in vitro* and *in vivo*. The results showed that inhibiting Sat1 could significantly inhibit ferroptosis by increasing the level of GSH and GPX4 protein expression and decreasing the level of LPO, lipid ROS generation, and TFR1 protein expression, thereby alleviating MIRI. Moreover, over-expression of Sat1 aggravated MIRI through the regulation of ferroptosis.

GO and KEGG analysis identified the MAPK signaling pathways as potential signaling pathways through which Sat1 regulates MIRI. The MAPK signaling pathway is a key signaling transduction axis that regulates gene expression of eukaryotic cells and plays a role in various cellular processes, including cell proliferation, differentiation, metabolism, angiogenesis, and stress responses (Zheng et al., 2020). To date, four members of the MAPK family have been identified, namely p38 MAPK, ERK, JNK/stress-activated protein kinase, and ERK5. Numerous studies have confirmed the vital role of MAPK in various diseases, including cardiovascular disease (Zhou et al., 2022), cancer (Lee et al., 2020), and diabetes mellitus (He et al., 2021). In the context of MIRI, it has been reported that the loss of DUSP1 can exacerbate cardiac injury by promoting cytochrome c release and mitochondrial permeability transition pore (mPTP) opening through JNK activation (Jin et al., 2018). Isoflurane has been shown to protect against MIRI in rats by inhibiting the phosphorylation of p38 MAPK, which is increased in MIRI and contributes to cardiac dysfunction (Zhou et al., 2019). The MAPK/ERK signaling pathway plays an important protective role in MIRI through various mechanism, including inflammation (Che et al., 2019), apoptosis (Ren et al., 2019) and endoplasmic reticulum stress (Su et al., 2021). Various signaling pathway have been studied in ferroptosis and MIRI. Liu et al. demonstrated that pachymic acid can inhibit ferroptosis of cardiomyocytes through activation of the AMPK pathway, thereby alleviating myocardial I/R injury in mice, while AMPK inhibitor obviously abolished the effect of pachymic acid on ferroptosis (Liu et al., 2024). Wang et al. found that FOXN4 can regulate MIRI through HIF-1α/MMP2-mediated ferroptosis (Wang et al., 2023). Zhao et al. showed that Zhilong Huoxue Tongyu Capsule (ZL) exerted a protective effect against MI/RI by inhibiting ferroptosis via PI3K/AKT pathway, and PI3K/AKT inhibitor reversed the anti-ferroptosis effects of ZL, thereby promote MIRI (Zhao et al., 2024). Jin et al. revealed that Oroxylin A alleviates myocardial ischemia-reperfusion injury by suppressing ferroptosis via activating the DUSP10/MAPK-Nrf2 pathway (Jin Y. et al., 2024). Chen et al. found that ERK, p38 MAPK and JNK pathways were significantly activated after MIRI, and inhibiting the phosphorylation of ERK, p38 MAPK and JNK could alleviate MIRI by inhibiting ferroptosis (Chen W. et al., 2023). Moreover, it is demonstrated that ROS-JNK/MAPK pathways mediated the protective effect of Salvianolic Acid B on MIRI through regulation of ferroptosis (Jin B. et al., 2024). Compare to other signaling pathway, MAPK pathway seems be of more concern in ferroptosis and MIRI. Therefore, we focused on the role of MAPK pathway, and found that MAPK/ERK pathway was significantly activated in OGD/R, and inhibition of Sat1 suppressed the phosphorylation of p-ERK. Moreover, rescue experiments demonstrated that MAPK/ERK pathway mediated the effect of Sat1 on ferroptosis. These results revealed that Sat1 play a role in MIRI through regulation of ferroptosis by MAPK/ERK pathway.

Moreover, GO analysis indicated that Sat1 was associated with transcriptional process. Thorough search of four databases, Sox2 was identified as potential transcription factor of Sat1. Current studies have reported that Sox2 can participate in the regulation of cancer proliferation, migration, invasion and metastasis through transcriptional regulation and other gene expression. It is closely related to the prognosis of cancer patients (Quintanal-Villalonga et al., 2023; Peng et al., 2022). However, no studies have reported its role in MIRI, and whether it plays a transcriptional regulatory role in the MIRI process and affects the expression of target genes needs to be further studied. In this study, it was found that Sox2 expression was downregulated in OGD/R. Moreover, Sox2 can suppress Sat1 expression. Therefore, during MIRI, decreased Sox2 expression may contribute to the increased expression of Sat1. However, what caused decreased Sox2 expression and what specific regulatory role it plays in ferroptosis and MIRI remain to be further studied.

There exist some limitations in this study. Firstly, there lack of Sat1 inhibitor or knockout experiments in animals, which may contribute to full understanding the role of Sat1 in ferroptosis and MIRI. Besides, pathway-specific inhibitors or agonists as control groups were not used to directly validate the role of MAPK/ERK pathway, which could exclude its own effects on H9c2 cells. If these issues above were finished in this study, which will make the results of this study more comprehensive.

In conclusion, our study provides evidence that Sat1 inhibition can alleviate MIRI by regulating ferroptosis through MAPK/ERK pathway, and Sat1 expression was negatively regulated by Sox2 at the transcription level. Hence, targeting Sat1 as a therapeutic approach could hold promise in the treatment of MIRI.

## Data Availability

The data used in this study are available from the corresponding author upon reasonable request.

## Abbreviations

MIRI, myocardial ischemia-reperfusion injury; LDH, lactic dehydrogenase; ROS, reactive oxygen species; DEGs, Differentially expressed genes; SAT1, spermidine/spermine N1-acetyltransferase 1; TFR1, transferrin receptor 1; GPX4, glutathione peroxidase 4; OGD/R, oxygen and glucose deprivation/reoxygenation; SD, Sprague–Dawley; LAD, left anterior descending; HE, hematoxylin and eosin; TTC, 2,3,5-triphenyltetrazolium chloride; TEM, transmission electron microscope; CCK-8, Cell Counting Kit-8; LDH, lactate dehydrogenase; GSH, glutathione; LPO, lipid peroxidation; GO, Gene Ontology; BP, biological process; CC, cellular component; MF, molecular function; KEGG, Kyoto Encyclopedia of Genes and Genomes; CTD, Comparative Toxicogenomics Database.
