# Supplementary figures and images for: Inhibition of Sat1 alleviates myocardial ischemia-reperfusion injury through regulation of ferroptosis via MAPK/ERK pathway
Source: Front Pharmacol. 2024 Nov 13;15:1476718. doi: 10.3389/fphar.2024.1476718 (PMC11599858; doi:10.3389/fphar.2024.1476718)

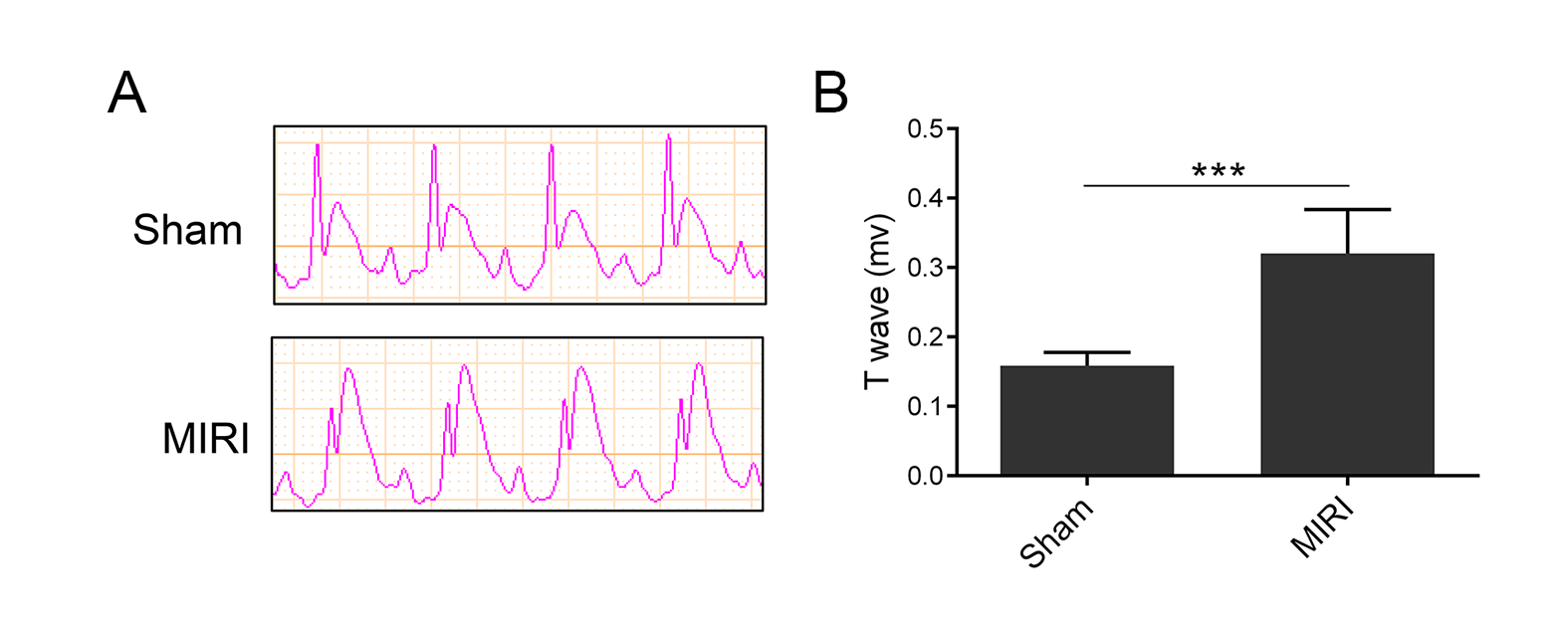

Supplement: Supplementary file 1 [file Image1.TIF]
